# HIV-1 functional cure: will the dream come true?

**DOI:** 10.1186/s12916-015-0517-y

**Published:** 2015-11-20

**Authors:** Chao Liu, Xiancai Ma, Bingfeng Liu, Cancan Chen, Hui Zhang

**Affiliations:** Institute of Human Virology, Zhongshan School of Medicine, Sun Yat-sen University, Guangzhou, 510080 China; Key Laboratory of Tropical Disease Control of Ministry of Education, Zhongshan School of Medicine, Sun Yat-sen University, Guangzhou, 510080 China

**Keywords:** cART, Functional cure, Genetic engineering, HIV-1, Latency reservoir, Shock and kill, Permanent silencing, Sterilizing cure

## Abstract

The reservoir of human immunodeficiency virus type 1 (HIV-1), a long-lived pool of latently infected cells harboring replication-competent viruses, is the major obstacle to curing acquired immune deficiency syndrome (AIDS). Although the combination antiretroviral therapy (cART) can successfully suppress HIV-1 viremia and significantly delay the progression of the disease, it cannot eliminate the viral reservoir and the patient must continue to take anti-viral medicines for life. Currently, the appearance of the ‘Berlin patient’, the ‘Boston patients’, and the ‘Mississippi baby’ have inspired many therapeutic strategies for HIV-1 aimed at curing efforts. However, the specific eradication of viral latency and the recovery and optimization of the HIV-1-specific immune surveillance are major challenges to achieving such a cure. Here, we summarize recent studies addressing the mechanisms underlying the viral latency and define two categories of viral reservoir: ‘shallow’ and ‘deep’. We also present the current strategies and recent advances in the development of a functional cure for HIV-1, focusing on full/partial replacement of the immune system, ‘shock and kill’, and ‘permanent silencing’ approaches.

## Introduction

Human immunodeficiency virus type 1 (HIV-1) is the causative pathogen of acquired immune deficiency syndrome ( AIDS), which has long been a global health concern. Combination antiretroviral therapy (cART), also known as highly active antiretroviral therapy (HAART), is the use of a combination of three or more antiretroviral drugs to suppress HIV-1 to an undetectable level in the blood plasma. It is one of the major medical successes of the 20th century. It effectively suppresses viral replication, improves the immune function, and significantly decreases the morbidity and mortality of AIDS. However, cART has many limitations: (i) it cannot eradicate the latent HIV-1 reservoir; (ii) the patient must maintain a lifelong treatment regimen, otherwise the viremia will rapidly rebound; (iii) it has toxic effects that some patients cannot tolerate; and (iv) it involves considerable expense. Therefore, the development of novel treatment methods that are not subject to these limitations is a massive requirement worldwide.

Ever since cases of HIV-1 infection were first reported in 1981, it has become a dream to cure the HIV-1 infection. There could be two categories of cure for HIV-1: a ‘sterilizing’ cure and a ‘functional’ cure. In most virus-induced diseases, the term ‘cure’ refers to a ‘sterilizing’ cure, which indicates that no trace of virus remains in the body. In 2009, the case of the ‘Berlin patient’ provided evidence that complete elimination of HIV-1 is possible [1]. After receiving whole-body irradiation and successful transplantation of bone marrow from a *CCR5* delta32 (Δ32) homozygous donor, the ‘Berlin patient’ reached what was thought to be a ‘sterilizing’ cure, given that no residual viruses were found several years after discontinuation of cART [2]. This case greatly encouraged enthusiasm for the development of a ‘sterilizing’ cure, until the case of ‘Boston patients’ appeared. Two patients suffering from both AIDS and lymphoma received a wild-type-*CCR5*(+) allogeneic hematopoietic stem cell transplantation (HSCT) in Boston. Although the levels of plasma viral RNA and the proviral DNA in peripheral blood mononuclear cells (PBMCs) remained undetectable after transplantation, viruses appeared again in their blood after cART was interrupted for 12 or 32 weeks, respectively [3]. The case of the ‘Mississippi baby’ was also a disappointment. This baby, whose mother had an untreated HIV-1 infection, received cART at 30 hours of age. The baby girl ceased cART treatment at 18 months of age. Surprisingly, the proviral DNA in PBMCs, the plasma viral RNA, and HIV-1 antibodies remained undetectable in her body when she was retested at 30 months [4]. In 2013, the girl’s doctors announced that she may have been cured and it seemed that the early treatment of HIV-1-infected babies could be a feasible strategy for curing them of HIV-1 infection. Unfortunately, plasma viral RNA and HIV-1 antibodies were detected in her blood when she was 4 years old [5].

As the ‘sterilizing’ cure for HIV-1 is difficult to achieve, a ‘functional’ cure for HIV-1 has become a global research priority. A ‘functional’ cure for HIV-1 is defined as a long-term host-mediated control of viral replication and remission of the symptoms of HIV-1 infection in the absence of antiretroviral therapy, even if replication-competent viruses remain in the body [6]. A rare group of individuals, termed ‘elite controllers’, spontaneously maintain plasma viremia to levels below the limits of clinical detection without cART treatment [7]. The mechanisms by which elite controllers maintain their viral loads as undetectable could involve several genetic traits. Over-representation of certain MHC class 1 alleles in elite controllers, such as class 1 HLA-B*57 and HLA-B*27 alleles, has a strong correlation with HIV-1 control capability [8, 9]. Potent HIV-1-specific CD8^+^ T cell response, such as the production of more cytolytic proteins and pro-inflammatory cytokines, can be found in elite controllers [10, 11]. The enrichment of specific natural killer (NK) cell immunoglobulin-like receptors (KIRs), such as KIR3DS1 and KIR3DL1, could also be a mechanism of elite control [12]. Furthermore, neutralizing antibodies via antibody-dependent cell-mediated cytotoxicity (ADCC) could also contribute to viral control, even though there have been conflicting results regarding the correlation between increased ADCC activity and elite controllers [13, 14]. Higher-degree DNA methylation in HIV-1 promoter was found to account for the delayed disease progression in elite controller [15]. High-level expression of specific host restriction factors (for example, Schlafen 11) have also been found in elite controllers [7]. Collectively, the elite controllers serve as a natural model for the ‘functional’ cure of HIV-1 and the mechanisms underlying the phenomenon could provide a road map for the control of HIV-1. Although efforts to pursue both ‘functional’ and ‘sterilizing’ cures are in progress, it seems that a ‘functional’ cure for HIV-1 is more feasible than a ‘sterilizing’ cure.

## HIV-1 latency is the major obstacle to achieving a cure

HIV-1 latency is a silent state in which the infected cells harbor intact HIV-1 genomes without generating viral particles. It can be divided into two forms: (i) pre-integration latency, which exists in a form with incomplete reverse transcripts or a form with un-integrated viral DNAs and has a short half-life lasting from one day to several weeks [16]; and (ii) post-integration latency, which is the major form of the viral reservoir and occurs after the infected activated CD4^+^ T cells reverse back to a resting state [17]. Because the resting CD4^+^ T cells that harbor HIV-1 proviruses are mostly long-lived central memory cells (T_CM_) and the integrated viral genome is quite stable, it has been estimated that the HIV-1 reservoir has a half-life of 44 months and needs almost 73.4 years to be completely purged [18]. Recent work has indicated that stem cell-like memory CD4^+^ T cells (T_SCM_) are also part of the HIV-1 reservoir [19]. In addition to resting CD4^+^ T cells, other cellular reservoirs that might exist include monocytes, macrophages, dendritic cells, hematopoietic progenitor cells (HPCs), and microglial cells [20, 21].

The mechanisms underlying the latency of HIV-1 have been studied extensively over the past few decades [22, 23]. Recent studies have expanded the knowledge base regarding many factors that might affect the reservoir, including epigenetic modifications, integration sites, and posttranscriptional regulations. One line of evidence indicated that epigenetic modifications are accountable for the molecular mechanism of HIV-1 latency [24]. Both histone deacetylase (such as HDAC-1 and HDAC-2) and histone methyltransferases (such as G9a, Suv39H1, HP1 gamma, GLP, and EZH2) facilitate the silencing of HIV-1 proviruses [25–29]. Although DNA methylation can downregulate gene expression and therefore plays a role in HIV-1 latency, there is also some disagreement regarding the correlation between the amount of CpG methylation in HIV-1 long terminal repeat (LTR) and HIV-1 latency [30, 31]. Earlier this year, a report showed a proportion of HIV-1 viruses to be silenced immediately upon invading the target cells in an *in vitro* model. Some actively transcribed proviruses also underwent slow inactivation [32]. The sudden silencing and time-dependent LTR inactivation were found to be positively correlated with polycomb repressive complex 2 (PRC2)-mediated H3K27 trimethylation, which is a repressive histone modification [32]. In addition, the orientation of HIV-1 promoter integrated into host genes has been reported to affect HIV-1 expression, termed ‘transcriptional interference’ (TI) [33, 34]. TI caused by elongation complex reading through HIV-1 promoter facilitates HIV-1 latency when viral DNA integrates into the introns of active genes with the same promoter orientation as host genes [34]. However, a conflicting result has shown that TI caused by read-through only occurs when the HIV-1 promoter was in an orientation opposite to that of the host genes. When both promoters were in the same orientation, HIV-1 gene expression could be promoted [33]. Besides, low-level expression of a HIV-1-encoded microRNA targeting the TATA-box region of viral promoter also plays a role in HIV-1 latency [35]. Posttranscriptional regulation is another molecular mechanism of HIV-1 latency. Host-derived noncoding RNAs (ncRNAs) regulate HIV-1 latency by affecting viral mRNAs [36, 37] or the expression of the host genes that are hijacked as HIV-1 co-factors [38].

It has been shown that many latently infected T cells undergo clonal expansion in individuals treated with cART [39, 40]. Some integration hotspots have been found in many of the genes in clonally expanded T cells, such as *megakaryoblastic leukemia 2* (*MKL2*) and *basic leucine zipper transcription factor 2* (*BACH2*). As both *MKL2* and *BACH2* are the genes closely related to cell proliferation, the activation of those genes by viral integration or transcription could account for the persistence of the infected cells [39–41]. In addition, the maintenance of the CD4^+^ T_CM_ viral reservoir is driven by T cell survival signal and low-level antigen-driven proliferation [42]. In contrast, the transitional memory CD4^+^ T cells (T_TM_), which are the major reservoirs in aviremic individuals, are maintained by interleukin-7 (IL-7)-driven homeostatic proliferation [42]. These two cellular mechanisms can explain why clonally-expended latently infected cells are frequently detected in individuals treated with cART [39–41].

The frequency of latently infected resting memory CD4^+^ T cells was once thought to be 1/10^6^ [43]. However, it has recently been found that, in addition to these inducible replication-competent proviruses, large quantities of non-inducible proviruses have intact genomes and normal LTRs from patients on cART [44]. A statistical model showed the size of the latent reservoir to be 60 times larger than previously estimated. Among the replication-competent proviruses, although only 1 % of replication-competent viruses can be recovered from the PBMCs by latency-reversing agents (LRAs), 11.7 % of the non-inducible proviruses have intact genomes and normal LTRs [44]. Even experiencing several rounds of stimulation, the proviruses in these cells were still silenced [44]. Conversely, the insufficiency of HIV-1-encoded proteins may be involved in the formation of different reservoirs. HIV-1 Tat, which is a transactivator of transcription, undergoes various modifications during its regulatory circuit [45–48]. Certain modifications may promote its decay, which leaves the proviruses without enough Tat for transcription. Based on the stochastic gene expression model of HIV-1 Tat fluctuation, the Tat feedback circuit is sufficient to control HIV-1 latency, which is independent of the cellular state [49]. The proviruses may be silenced in the cells that do not have enough Tat, even if that cell has reached its maximum activation. Therefore, if enough Tat protein accumulates, the silenced viral reservoir could be activated by LRAs.

These new findings suggest that two levels of viral reservoir may exist: the ‘shallow’ viral reservoir and the ‘deep’ viral reservoir. The ‘shallow’ viral reservoir consists of the infected cells containing inducible proviruses, most of which could be reactivated by LRAs. In contrast, the infected cells harboring non-inducible proviruses constitute the ‘deep’ viral reservoir, which cannot be activated by current LRAs including the stimulating agents for lymphocyte proliferation. However, the formation of ‘deep’ viral reservoirs has not been fully understood. It could be due to the special HIV-1 integration sites, the special epigenetic regulations including DNA methylation or histone modifications, the spatial organization of the genomes, the mediation of the interactions of distal elements, or special cell status, etc. [31, 32, 39–41, 44, 49]. More effort is needed to clarify these mechanisms and further, more *in vivo* evidence is required for the classification of these two reservoirs.

## Full or partial replacement of the immune system through genetic modification: strategy 1 for the functional cure of HIV-1

While the case of the ‘Berlin patient’ resulted in a successful cure of HIV-1, the low frequency of matching both *CCR5*Δ32 and HLA genotypes for allogeneic bone marrow transplantation and the scarcity of patients suffering from both HIV-1 infection and lymphoma/leukemia must be considered. Thus, the replacement of all or part of the immune system through genetic engineering to produce CD4^+^ T cells resistant to HIV-1 infection is a more feasible strategy. Since *CCR5* was identified as the primary HIV-1 co-receptor and the *CCR5*Δ32 CD4^+^ T cells were found to be resistant to HIV-1 infection, the reduction or elimination of *CCR5* expression have been pursued [50, 51]. Several genetic approaches have been utilized to knock down *CCR5* in primary immune cells, including the use of ribozymes [52], single-chain intracellular antibodies [53], trans-dominant co-receptor mutants [54], or RNA interference (RNAi) [55]. However, these strategies do not silence *CCR5* expression permanently. To reach this goal, some gene editing technologies, including zinc finger nucleases (ZFNs) and transcription activator-like effector nucleases (TALENs), have been used to knock out the *CCR5* gene [56, 57]. Several clinical trials using autologous infusions of *CCR5*-modified CD4^+^ T cells are currently under way. They are listed at ClinicalTrials.gov under ID numbers NCT00842634, NCT01252641, and NCT01044654 [58]. However, some T lymphocyte-tropic virus strains (X4 and R5X4) utilize CXCR4 as another co-receptor and these viral strains are found in approximately 50 % of late-stage HIV-1-infected individuals, supporting the therapeutic requirement targeting CXCR4 [59, 60]. After inhibition or disruption of CXCR4 in primary lymphocytes using siRNA [61] or ZFNs [58], the CD4^+^ T lymphocytes became capable of resisting CXCR4-tropic HIV-1 infection. To fully protect cells from HIV-1 infection, both *CXCR4* and *CCR5* genes must be disrupted in CD4^+^ T lymphocytes. These double co-receptor negative cells were resistant to both CCR5- and CXCR4-tropic HIV-1 infection in a humanized mouse model [62], suggesting that this strategy is a reasonable approach in HIV-1 functional cure. Recently, CRISPR/Cas9 system as a novel method of gene editing was used to disrupt *CCR5* in T cell lines, primary CD4^+^ T cells, or induced pluripotent stem cells (iPSCs) to make them or their progeny cells resistant to HIV-1 infection [63–65]. Moreover, the CRISPR/Cas9 system could also be used as a long-lived intracellular defender to protect cells from HIV-1 infection. Once the HIV-1 entered the cells, the HIV-1-specific CRISPR/Cas9 could quickly induce lethal mutations in the incoming viral DNAs or even completely excise them [66, 67].

## Shock and kill: strategy 2 for the functional cure of HIV-1

As latently infected resting CD4^+^ T cells do not expose viral antigen, the immune system cannot recognize and destroy them. For this reason, the ‘shock and kill’ (also known as ‘kick and kill’) strategy has been extensively discussed. After reactivating latent viruses from the reservoir, the immune surveillance system will recognize and eradicate these HIV-1-expressing cells in various ways, including cytotoxic T lymphocyte (CTL) response or ADCC.

### Shock

Several methods of reactivating latent HIV-1 have been developed. Anti-CD3 monoclonal antibody, interleukin-2 (IL-2), and tumor necrosis factor-α (TNF-α) were used to activate HIV-1 replication from the latently infected cells. However, given that the massive activation of CD4^+^ T cells by these general lymphocyte activators involves a high risk of systemic induction of proinflammatory cytokines, these strategies are currently not used clinically. It has also been demonstrated that IL-7 can activate HIV-1 latency [68]. However, clinical trials of IL-7 administration in HIV-1-infected individuals treated with cART have indicated that IL-7 can mediate survival and expansion of latently infected memory CD4^+^ T lymphocytes and promote HIV-1 persistence [69].

Apparently, more specific LRAs are needed to specifically activate HIV-1 latency. One feasible strategy is to trigger the activity of NF-κB, a major host transcription factor, in cells latently infected with HIV-1 for viral reactivation. Some protein kinase C (PKC)-NF-κB pathway activators, such as prostratin and bryostatin-1, can reactive viruses *in vitro* [70, 71]. As prostratin induces potent upregulation of the transcription of several cytokines [71], its cytokine-related toxicity should be carefully evaluated in a clinical trial. Bryostatin-1, which has already been evaluated in several clinical trials for cancer, is currently undergoing clinical trial for HIV-1 latency (NCT02269605). Conversely, some histone deacetylase inhibitors (HDACis), which can keep more histones in a state of acetylation and loosen the condensed chromatin structure, have been developed as LRAs. Valproic acid (VPA), which has been approved to treat neuropsychiatric conditions, could induce viral reactivation in resting CD4^+^ T cells from HIV-1-infected patients on cART [72]. A proof-of-concept study in four volunteers showed that the combination therapy with VPA and intensified cART led to a decrease in the number of latently infected cells in three of four patients [73]. However, subsequent studies did not support these data [74–76]. Vorinostat, also called suberoylanilide hydroxamic acid (SAHA), significantly increases HIV-1 transcription in an *ex vivo* HIV-1 latency model [77]. It has already been in phase I and II clinical trials. A single-dose trial of vorinostat demonstrated that the administration of the drug could reactivate HIV-1 latency *in vivo* [78]. Other potent HDACis, such as romidepsin (FK288) and panobinostat (LBH589), have entered clinical trials [79, 80]. After romidepsin infusions, plasma HIV-1 RNA was found to significantly increase at multiple post-infusion time points in five out of six HIV-1-infected patients [80]. In a phase I/II clinical trial, panobinostat reactivated HIV-1 latency *in vivo* effectively but did not reduce the number of latently infected cells, indicating that this agent may need to be combined with other LRAs to affect the HIV-1 reservoir [79]. Although the reactivation potency of givinostat (ITF2357) and belinostat (PXD101) have been demonstrated in cell lines latently infected with HIV-1, their effects on HIV-1 latency need to be evaluated *in vivo*.

In addition, as histone methylation plays a major role in chromatin-mediated repression of the HIV-1 promoter, H3K9 histone methyltransferase inhibitors (HMTis), including BIX01294, chaetocin, and 3-deazaneplanocin A (DZNep), have been used to induce HIV-1 reactivation in latently infected resting CD4^+^ T cells or Jurkat T cells [27, 28, 81]. It is expected that the clinical trials of HMTis for HIV-1 latency could be attempted in the future. Interestingly, disulfiram, which has been used to treat alcoholism for decades, was identified as an HIV-1 LRA in an *in vitro* latency model [82]. In a pilot study, disulfiram was found to be safe and led to a transient increase in plasma HIV-1 RNA in a subset of individuals [83]. The possible mechanism of disulfiram on HIV-1 transcription is to reduce phosphatase and tensin homolog (PTEN) expression level, which results in activation of the Akt signaling pathway [84]. However, the administration of disulfiram alone or in combination with a modified vaccinia Ankara-based HIV-1 vaccine (MVA-B) is unable to reduce the size of the latent reservoir [83, 85, 86]. Furthermore, it has been demonstrated that bromodomain-containing protein 4 (BRD4) can interact with the P-TEFb complex. BRD4 is a member of the bromodomain and extra terminal domain (BET) family and a well-conserved transcriptional regulator that recognizes and binds acetyl-lysine residues [87, 88]. By affecting BRD4 and P-TEFb interaction, BET bromodomain inhibitors, such as JQ-1 and I-Bet, potently reactivate HIV-1 latency [89, 90]. Recently, two groups showed that PKC agonists in combination with BET bromodomain inhibitors or histone deacetylase inhibitors robustly induced the reversal of HIV-1 latency *in vitro* and *ex vivo* as effectively as positive controls stimulated with PMA plus ionomycin or anti-CD3 plus anti-CD28 antibodies [91, 92]. The potent effect of LRA combinations indicates that co-administration of different types of LRAs could be a suitable means of reducing the size of viral reservoirs.

### Kill

After latent viruses are reactivated by the LRAs, the immune system could eradicate the virus-producing cells. However, recent data indicated that CD8^+^ T cells in HIV-1-infected individuals on cART cannot eliminate latently infected cells even after successful reactivation [93]. Therefore, the recovery of potent antiviral function of the immune system is required for the ‘kill’ strategy. A role for IFN-α-mediated clearance of HIV-1-infected cells is supported by several studies in which the expression of perforin and granzyme in NK and CD8^+^ T cells from patients with HIV-1 infection or melanoma was increased by IFN-α immunotherapy [94, 95]. The administration of pegylated IFN-α-2A to patients on cART was found to be associated with decreased HIV-1 integration and suppression of viral replication [96]. Importantly, checkpoint blockage, which is currently used for cancer immunotherapy, has been examined for HIV-1 treatment. Programmed cell death protein 1 (PD-1), a T cell surface receptor, is highly expressed on latently infected CD4^+^ or CD8^+^ T cells in patients treated with cART. High expression of PD-1 on HIV-1-specific CD4^+^ T cells prevents the activation of T cell receptors (TCRs) and leads to the inhibition of proliferation and secretion of diverse cytokines, resulting in the failure of immune surveillance that recognizes and kills the reactivated HIV-1-infected CD4^+^ T cells [97–99]. Conversely, PD-1 blockage leads to the improvement of HIV-1-specific CD8^+^ T cell proliferation and cytokine secretion [100, 101]. In the SIV-macaque model, anti-PD-1 antibodies restored the function of SIV-specific CD4^+^ and CD8^+^ T cells [102]. Therefore, anti-PD-1 antibodies could be employed to enhance the CTL response of HIV-1-specific CD8^+^ T cells.

Autologous adoptive transfer of HIV-1-specific CD8^+^ T cells is another way to directly enhance the CTL response of patients on cART. This strategy has been widely used in cancer immunotherapy [103–105]. Given that CD8^+^ T_CM_ cells possess the ability of self-renewal and maintain robust responses over time, HIV-1-specific CD8^+^ T cells from patients on cART can be expanded *ex vivo* and persist *in vivo* for more than 84 days, suggesting that more methods targeting CD8^+^ T_CM_ cells in patients treated with cART could provide an expanded and long-term immune response [106]. Although CTL response plays a significant role in the clearance of reservoir, HIV-1 can quickly mutate to evade the CTL reorganization. Therefore, the predominance of CTL-resistant viruses in the reservoir is a major barrier to viral eradication. A systematic investigation of CTL escape variants in reservoirs has recently been performed. Results showed that some CTLs that recognize unmutated epitopes can exist in every tested patient. These broad-spectrum CTLs have potent antiviral activity and can eliminate target cells both *in vitro* and in the humanized mice generated with the bone marrow cells from HIV-1-infected patients [107]. Therefore, strategies utilizing this broad-spectrum viral-specific CTL response could be further developed for the eradication of the HIV-1 reservoir.

Some genetic modification strategies have been used to enhance the specific response of CD8^+^ T cells to HIV-1-infected cells. To achieve powerful and sustained CTL responses by enhancing TCR binding affinity, artificial TCR (aTCR) was generated by special phage display technology [108]. Two groups have reported that CD8^+^ T cells transduced with HIV-1 Gag-specific SL9 aTCR could lyse A2-SL9-expressing cells and effectively controlled wild-type and mutant strains of HIV-1 *in vitro* and *in vivo* [109, 110]. However, given that the off-target toxicity of MAGE-A3-specific aTCR for myeloma and melanoma in a clinical trial caused the death of some patients [111], a clinical trial of HIV-1-infected patients, NCT00991224, was suspended before any participant was infused with A2-SL9-specific aTCR-transduced CD8^+^ T cells [112]. Therefore, the safety and specificity of high affinity TCR-modified CD8^+^ T cells need to be carefully re-evaluated.

Recently, modifying CD8^+^ T cells with chimeric antigen receptors (CARs) has become a hot topic in cancer research. CARs are generated by combining an extracellular antigen-binding domain with intracellular T cell activation domains. The extracellular antigen-binding domain could be the antigen-binding domain of the antibody or natural molecular marker such as CD4, which can interact with HIV-1 gp120. The most common intracellular domain is the CD3 zeta chain, which is the signal transduction machinery for T cell receptors [113]. Modifying CD8^+^ T cells with these CARs allows T cells to retarget the immune system in a high-affinity, TCR-independent, and MHC-unrestricted manner. The clinical usage of CARs for cancer adoptive therapy has been shown to be safe and highly effective, mediating remission in approximately 80 % of acute lymphocytic leukemia patients [114]. CAR-T cells can survive in the human body for more than 11 years after infusion [115]. CD8^+^ T cells modified with HIV-1-targeting CARs lysed HIV-1 gp120-expressing cells specifically *in vitro* [116, 117]. However, the antiviral efficacy of first-generation CAR-T *in vivo* was modest [118]. More recently, a new generation of CARs has been developed by including different parts of the signaling molecules, such as 4-1BB and/or CD28, to promote cell-mediated cytotoxicity, proliferation, and survival of the modified CAR-T cells [119–121]. Additionally, to prevent the CD4-CARs from functioning as HIV-1 entry receptors, some novel CD4-CARs have been designed by co-expressing with HIV-1 fusion inhibitor (for example, maC46, an extended form of enfuvirtide) or linking CD4 to a single chain variable fragment of the 17b human monoclonal antibody, termed ‘17b scFv’ [122, 123]. Thus, the new generations of HIV-1-specific CAR-T cells should be used to enhance HIV-1-specific CTL response and their clinical usage could eventually be seen.

More recently, several studies have demonstrated that some HIV-1-specific broadly neutralizing monoclonal antibodies (bNAbs) (for example PGT121, VRC01, and VRC03) can block cell-cell transmission of HIV-1 and suppress viral replication in primary CD4^+^ T cells from patients on cART *in vitro* and also control viremia in infected humanized mice or rhesus macaques effectively [124–128]. This year, 3BNC117, a potent CD4-binding site antibody entered clinical trial [129]. As 3BNC117 is well tolerated and effective in controlling HIV-1 viremia, bNAb therapy can be explored as a new strategy for HIV-1 functional cure. Given that HIV-1 can quickly mutate to escape from a single antibody, the combinations of bNAbs have been tried in a HIV-1-infected humanized mice model [124, 126]. These bNAb combinations have shown long-term half-life in blood plasma for an average of 60 days and resulted in a significant reduction in both viremia and cell-associated HIV-1 DNA. Moreover, bNAbs plus a combination of LRAs also showed a significant decrease in viral reservoir in humanized mice [130]. Thus, the combinations of LRAs and bNAbs could be developed as a therapeutic modality for eradicating viral reservoir in clinic.

## Render HIV-1 permanently silent: strategy 3 for the functional cure of HIV-1

Until now, all of the cure strategies have been aimed at reducing or eradicating the viral reservoir. However, replacing all or part of the immune system through genetic modifications can only render uninfected cells immune to HIV-1. The infected cells remain alive and may proliferate. The ‘shock and kill’ strategy is based on the prerequisite that the infected cells and the proviruses should be reactivated. As the current ‘shock’ agents cannot wake up HIV-1 proviruses in all the latently infected cells, it is unlikely that the reservoir of HIV-1 will be completely eradicated using these recent approaches.

Due to the non-inducible property of the ‘deep’ viral reservoir, the ‘permanent silencing’ approach could be considered as a suitable cure strategy. Unlike ‘shock and kill’ strategies, this approach focuses on silencing or locking the HIV-1 proviruses in cells. DNA hypermethylation of viral promoter and enhancer has been shown to be important to HIV-1 silencing [31]. The transcriptional gene silencing (TGS) siRNAs or short hairpin RNA (shRNA)-targeting NF-kB binding motifs in the HIV-1 LTR successfully suppressed the production of HIV-1 for up to one year [131, 132]. This suppression was found to be associated with CpG methylation within the 5′LTR and also achieved in NOJ mice reconstituted with human PBMCs [131–133]. Another group used mobilization-competent lentiviral vector to deliver small noncoding RNA targeting HIV-1 LTR. They successfully silenced HIV-1 for over one month without resistance mutation in TZM-bl cells. This TGS of HIV-1 was accomplished through increasing silent epigenetic modifications, such as histone and DNA methylation [134]. However, increasing viral burden results in the loss of the antiviral effect in primary CD4^+^ T cells [135]. Besides, their TGS-based antisense RNAs activated tumor suppressor p53. The delivery, efficiency, and safety of RNA-mediated TGS must be considered in future studies.

Alternatively, many therapeutic applications of CRISPR/Cas9 technologies for HIV-1 have been investigated [136]. In addition to its capability to disrupt CCR5 expression and break down the viral pre-integration complex (PIC) [63–65], CRISPR/Cas9 has been used to directly mutate the integrated proviruses in T cell lines. The latently integrated viral genome and viral replication were significantly disrupted [137]. Future work should determine whether the CRISPR/Cas9-mediated silencing can be applied to latently infected resting CD4^+^ T cells from cART patients.

However, these ‘permanent silencing’ strategies described above have some limitations in clinical application: (i) delivery difficulties *in vivo*; (ii) low efficiency of disrupting target gene(s); and (iii) uncertain safety issues because of genetic modifications. Thus, development of silencing small molecule compounds could be a feasible approach. Indeed, chlorate and guaiacol, which inhibit the sulfonation pathway, have been found to restrict HIV-1 reactivation through blocking viral transcription initiation [138]. One group synthesized and characterized several p300-HAT-specific inhibitors. These compounds can inhibit the acetylation of HIV-1-infected T cells and therefore silence HIV-1 [139]. To reach the goal of silencing HIV-1 transcription permanently, more epigenetic modifications around integrated proviruses need to be identified for developing more new inhibitors. The absence of HIV-1 Tat could render provirus ‘deeply’ silenced in the cell as described above. Screening the drugs directly inhibiting Tat can be another alternative strategy. Didehydro-Cortistatin A (dCA), an analog of the natural steroidal alkaloid cortistatin A, was found to suppress HIV-1 transcription through blocking the TAR binding domain of Tat [140]. It can block provirus reactivation in several cellular models of HIV-1 latency and CD4^+^ T cells isolated from aviremic individuals [141]. Long-term treatment of this compound can induce a nearly permanent silent state which is insensitive to current LRAs. Combinations of the inhibitors of HIV-1 Tat with cART may help to deeply silence the proviruses in the cells, delay the replenishment of reservoir, and finally shrink it. Another strategy to silence proviruses is to utilize cellular signal pathway inhibitors. Recently, INK128, one of the mTOR kinase inhibitors (TOR-KIs), was found to inhibit several steps of the HIV-1 lifecycle, including CCR5-mediated entry and the transcription of HIV-1 genes [142]. The suppression was also achieved in humanized mice over two weeks. Whether the TOR-KIs can silence HIV-1 through immunomodulatory mechanisms needs to be further studied.

## Conclusions

**Fig. 1:**
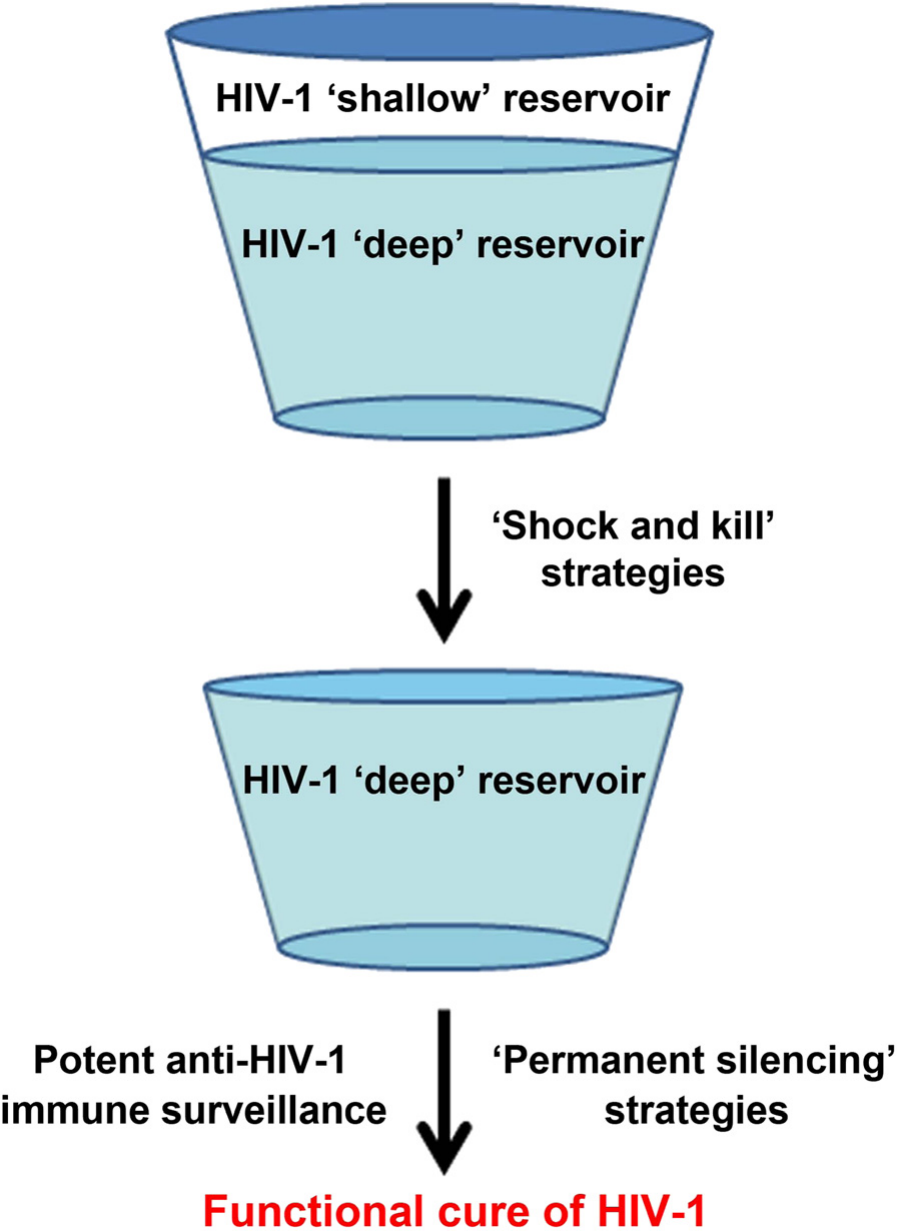
A feasible strategy for the functional cure of HIV-1. Firstly, ‘shock and kill’ strategies could be quite useful in kicking out the ‘shallow’ latent viruses and eliminating them. Then, ‘silencing’ strategies, which permanently inactivate the ‘deeply’ silenced viruses, accompanied by potent anti-HIV-1 immune surveillance, could subsequently be utilized to achieve the functional cure of HIV-1

Latent infection remains a major obstacle to curing HIV-1. Several strategies have been proposed and attempted, including full/partial replacement of the immune system, ‘shock and kill’, and ‘permanent silencing’. However, to date, most of these strategies are still in the experimental stage. None of the strategies under discussion can eliminate the reservoir completely and many challenges still remain. As given above, the viral reservoir can be categorized into the ‘shallow’ reservoir and the ‘deep’ reservoir (Fig. 1). The ‘shock and kill’ strategy can be very effective in the elimination of the ‘shallow’ reservoir by kicking viruses out of the cells and eliminating them. As the mechanisms by which the viral reservoir is established and maintained are complicated, the development of novel, effective, and specific LRAs and the combinations of LRAs functioning in different signal pathways could be required. In several studies, combinations of prostratin (PKC activator) and DNA methylation inhibitor, PKC agonists plus BET bromodomain inhibitors, HMTi and HDACi, or HDACi plus prostratin and TNF-α have been attempted and they have been shown to increase the activation of latently infected cells [91, 92, 143–145]. Considering some LRAs are capable of generally reactivating T cells, their toxicity and potential risk need to be evaluated and monitored closely.

**Fig. 2:**
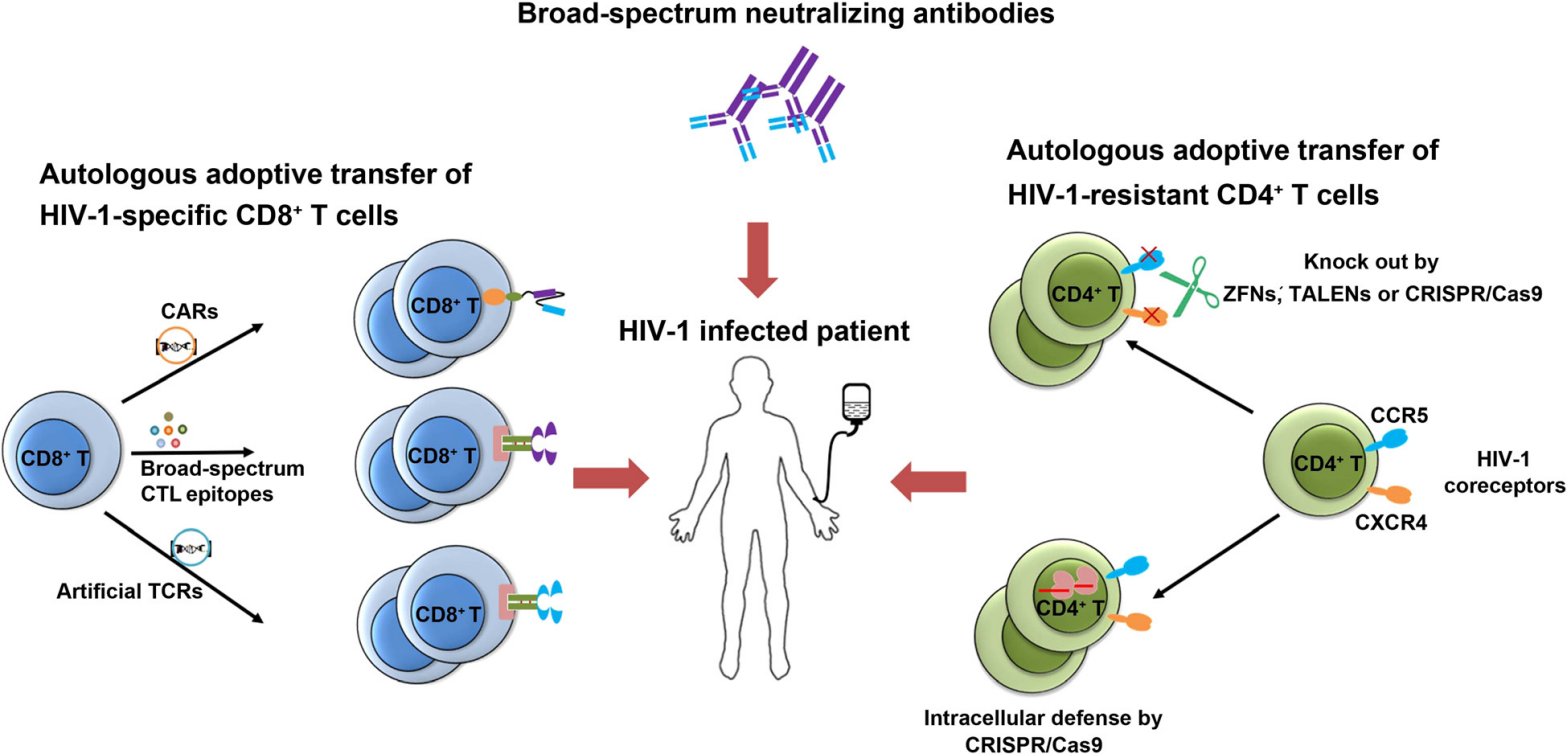
Strategies for recovery and optimization of anti-HIV-1 immune surveillance. The combination of bNAbs with autologous adoptive transfer of HIV-1-specific CD8^+^ T cells and/or HIV-1-resistant CD4^+^ T cells could be an effective therapeutic modality for the functional cure of HIV-1

The enhancement of anti-HIV-1 immune surveillance is a conventional choice for the ‘kill’ strategy. Based on the effective suppression of bNAbs against HIV-1 in animal models and clinical trial, combinations of bNAbs with LRAs and/or ‘kill’ approaches need to be further tested. However, given that bNAbs are expensive and are unlikely used lifelong, these approaches can only be used to eradicate the ‘shallow’ reservoir for a short time being. To build up a long-term anti-HIV-1 immune surveillance, cell therapy should be further developed. Previous data have demonstrated that the immune systems of patients on cART are not strong enough to eradicate reactivated CD4^+^ T cells [93]. For this reason, rebuilding immune surveillance in patients by autologous adoptive transfer of HIV-1-resistant CD4^+^ T cells and HIV-1-specific CD8^+^ T cells is a feasible strategy (Fig. 2). Novel gene editing techniques (such as CRISPR/Cas9 system, high affinity TCRs, and new generations of CAR-Ts) could be attempted in clinical trials. Additionally, more strategies pointing at CD8^+^ T cells from patients on cART, such as autologous adoptive transfer of *ex vivo* expanded HIV-1-specific CD8^+^ cells, could provide a strong and expanded CTL response. Furthermore, with the discovery of broad-spectrum viral-specific CTL response, autologous adoptive transfer of HIV-1-specific CD8^+^ T cells induced and educated by these broad-spectrum CTL epitopes could be developed as a therapeutic maneuver to promote the clearance of HIV-1-infected cells. Importantly, the selective expansion of HIV-1-specific CD8^+^ T_CM_*ex vivo* allows the T_CM_ with the long-term self-renewal ability to respond to and eradicate any reactivated infected cells. Therefore, the potent immune surveillance could persistently control viral replication without the continuation of cART (Fig. 2). As such, functional cure of HIV-1 for a long term, even for a lifelong, could be achieved.

To eliminate the ‘deep’ reservoir, highly effective and specific latency activators and combinations of LRAs from different pathways can be used to remove the ‘shallow’ reservoir and reduce the size of the ‘deep’ reservoir to the greatest extent. Thereafter, strategies such as the CRISPR/Cas9 system, RNAi, and silencing small molecule compounds could be utilized to inactivate these ‘deeply’ silenced viruses. In this case, this strategy should be used alongside the ‘kill’ strategies to enhance and maintain the immune surveillance (Fig. 1).

In summary, the ‘shallow’ and ‘deep’ reservoir classification can guide the selection of treatment modalities (Fig. 1). Further investigations on the mechanisms of HIV-1 latency are required for the development of new strategies to eradicate viral reservoir. It is also important to search for novel LRAs to shock out more genetically-diversified viruses and develop new immunotechnologies that could recognize and kill the virus-infected cells. Moreover, new genetic engineering methods capable of permanently silencing proviruses or even replacing the immune system entirely also need to be developed. Despite the failure of current strategies to eliminate or control HIV-1, the results still provide certain directions for further investigation of therapeutic interventions. Considering that the HIV-1 reservoir seems nearly impossible to be eradicated completely, the dream for ‘functional’ cure of HIV-1 should be the ultimate goal.

## Abbreviations

ADCC: Antibody-dependent cell-mediated cytotoxicity
AIDS: Acquired immune deficiency syndrome
aTCR: Artificial TCR
*BACH2*: *Basic leucine zipper transcription factor 2*
BET: Bromodomain and extra terminal domain
bNAb: Broadly neutralizing monoclonal antibody
BRD4: Bromodomain-containing protein 4
CAR: Chimeric antigen receptor
cART: Combination antiretroviral therapy
CTL: Cytotoxic T lymphocyte
dCA: Didehydro-Cortistatin A
DZNep: 3-Deazaneplanocin A
HAART: Highly active antiretroviral therapy
HDACi: Histone deacetylase inhibitor
HIV-1: Human immunodeficiency virus type 1
HMTi: Histone methyltransferase inhibitor
HPC: Hematopoietic progenitor cell
HSCT: Hematopoietic stem cell transplantation
IL-2: Interleukin-2
IL-7: Interleukin-7
iPSC: Induced pluripotent stem cell
KIR: Killer cell immunoglobulin-like receptor
LRA: Latency-reversing agent
LTR: Long terminal repeat
*MKL2*: *Megakaryoblastic leukemia 2*
ncRNA: Noncoding RNA
NK: Natural killer
PBMC: Peripheral blood mononuclear cell
PD-1: Programmed cell death protein 1
PIC: Pre-integration complex
PKC: Protein kinase C
PRC2: Polycomb repressive complex 2
PTEN: Phosphatase and tensin homolog
RNAi: RNA interference
SAHA: Suberoylanilide hydroxamic acid
shRNA: Short hairpin RNA
TALENs: Transcription activator-like effector nucleases
T_CM_: Central memory CD4^+^ T cells
TCR: T cell receptor
TGS: Transcriptional gene silencing
TI: Transcriptional interference
TNF-α: Tumor necrosis factor-α
TOR-KI: mTOR kinase inhibitor
T_SCM_: Stem cell-like memory CD4^+^ T cell
T_TM_: Transitional memory CD4^+^ T cell
VPA: Valproic acid
ZFN: Zinc finger nuclease
